# Attributable risks of hospitalizations for urologic diseases due to heat exposure in Queensland, Australia, 1995–2016

**DOI:** 10.1093/ije/dyab189

**Published:** 2021-09-11

**Authors:** Peng Lu, Guoxin Xia, Qi Zhao, Donna Green, Youn-Hee Lim, Shanshan Li, Yuming Guo

**Affiliations:** 1 Department of Epidemiology, School of Public Health and Management, Binzhou Medical University, Yantai, Shandong, China; 2 Department of Epidemiology and Preventive Medicine, School of Public Health and Preventive Medicine, Monash University, Melbourne, Victoria, Australia; 3 Department of Epidemiology, School of Public Health, Cheeloo College of Medicine, Shandong University, Jinan, China; 4 Climate Change Research Centre and ARC Centre of Excellence for Climate Extremes, University of New South Wales, Sydney, Australia; 5 Section of Environmental Health, Department of Public Health, Faculty of Health and Medical Sciences, University of Copenhagen, Copenhagen, Denmark

**Keywords:** Increasing temperatures, hospitalization, urologic disease, attributable risks, Queensland, Australia

## Abstract

**Background:**

Heat exposure is a risk factor for urologic diseases. However, there are limited existing studies that have examined the relationship between high temperatures and urologic disease. The aim of this study was to examine the associations between heat exposure and hospitalizations for urologic diseases in Queensland, Australia, during the hot seasons of 1995–2016 and to quantify the attributable risks.

**Methods:**

We obtained 238 427 hospitalized cases with urologic diseases from Queensland Health between 1 December 1995 and 31 December 2016. Meteorological data were collected from the Scientific Information for Land Owners—a publicly accessible database of Australian climate data that provides daily data sets for a range of climate variables. A time-stratified, case-crossover design fitted with the conditional quasi-Poisson regression model was used to estimate the associations between temperature and hospitalizations for urologic diseases at the postcode level during each hot season (December–March). Attributable rates of hospitalizations for urologic disease due to heat exposure were calculated. Stratified analyses were performed by age, sex, climate zone, socio-economic factors and cause-specific urologic diseases.

**Results:**

We found that a 1°C increase in temperature was associated with a 3.3% [95% confidence interval (CI): 2.9%, 3.7%] increase in hospitalization for the selected urologic diseases during the hot season. Hospitalizations for renal failure showed the strongest increase 5.88% (95% CI: 5.25%, 6.51%) among the specific causes of hospital admissions considered. Males and the elderly (≥60 years old) showed stronger associations with heat exposure than females and younger groups. The sex- and age-specific associations with heat exposure were similar across specific causes of urologic diseases. Overall, nearly one-fifth of hospitalizations for urologic diseases were attributable to heat exposure in Queensland.

**Conclusions:**

Heat exposure is associated with increased hospitalizations for urologic disease in Queensland during the hot season. This finding reinforces the pressing need for dedicated public health-promotion campaigns that target susceptible populations, especially for those more predisposed to renal failure. Given that short-term climate projections identify an increase in the frequency, duration and intensity of heatwaves, this public health advisory will be of increasing urgency in coming years.

Key Messages

- A 1°C increase in temperature was associated with a 3.3% (95% confidence interval: 2.9%, 3.7%) increase in hospitalization for urologic diseases during the hot season.
- People with renal failure were most sensitive to heat exposure.
- Males and the elderly (≥60 years old) showed stronger associations with heat exposure than females and younger groups, respectively.
- Nearly one-fifth of hospitalizations for urologic diseases were attributable to heat exposure in Queensland in the hot season.

## Introduction

The urologic system, composed of the kidneys, ureters, bladder and the urethra, is the main excretion system that regulates blood volume and the balance of electrolytes.^1^ Urologic diseases include diseases and disorders that affect the urinary system such as kidney failure, urolithiasis and urinary-tract infection. It is estimated that the annual burden of kidney diseases alone accounts for 5–10 million deaths worldwide.^2^ The number of global disability-adjusted life years (DALYs) attributable to kidney disease increased from 19 million in 1990 to 33 million in 2013.^3^ In Australia, the treatment of end-stage renal disease (ESRD) dialysis alone represented 13% of hospitalizations in 2016 and dialysis admissions continue to increase each year.^4^ The cumulative cost of treating ESRD from 2009 to 2020 was estimated to be between USD$8.6 and $9.4 billion.^5^

Recent international studies have identified heat exposure as a risk factor for urologic diseases in occupational settings.^6^^,^^7^ In Adelaide, Australia, a 1°C increase in daily temperature (including minimum, average, maximum temperatures) was associated with increased risks of hospital admissions with specific causes of urologic diseases (except for pyelonephritis) in the warm season of October to March during 2003–2014.^8^ Another study in Queensland, Australia, analysed the association between hourly temperature and hourly emergency-department visits for acute kidney injury in the five hottest months for the years 2013–2015. The results found that the effect of heat on acute kidney injury occurred in the same hour of heat exposure, with odds ratio (OR): 1.37; 95% confidence interval (CI): 1.10, 1.71.^9^ Queensland is a urologic disease hotspot where at least 12% of the population have signs of chronic kidney disease.^10^ This burden is likely to be an underestimate because more patients are likely to not even be aware that they may have these medical conditions due to their asymptomatic early stages.^11^ Queensland has a typical subtropical–tropical climate with hot, humid summers. Given the recent unprecedented extreme summer weather across Australia^12^ and the ongoing increase in heat extremes, these higher temperatures will inevitably bring more challenges to this population’s urologic systems. Given the significance in terms of human health and well-being, and the financial cost, it is surprising that there are limited existing studies that have examined the relationship between high temperatures and urologic disease, or estimated the attributable risk of the disease to heat exposure in Queensland. There is also a paucity of research that examines demographic, spatial and socio-economic characteristics that would enable an identification of susceptible populations to heat exposure.^13^

This study tests our hypothesis that high temperatures increase the risks of hospitalization for urologic diseases and examines the potential modifiers for this association during the hot season in Queensland between 1995 and 2016. We examined the effect modifiers of intrinsic factors of age and sex and extrinsic factors of local climate and socio-economic status. We stratified urologic diseases into cause-specific types.

## Methods

### Data collection

We obtained de-identified data on hospitalizations for urologic diseases from Queensland Hospital Admitted Patient Data Collection (QHAPDC), Queensland Health, from 1 December 1995 to 31 December 2016. The QHAPDC collects demographic data and clinical information on all admitted patients separated from both public and licensed private hospitals and private day surgeries in Queensland. Informed consent was not needed, as Queensland Health only provided de-identified data. The data included all urologic system diseases (primary diagnosis), date of admission, age, sex and postcode. We chose all the primary diagnoses of urologic diseases as our cases. The other diagnoses of urologic diseases, such as secondary diagnoses, were excluded. The diagnosis of urologic disease follows the International Classification of Diseases, Ninth Revision (ICD-9) codes: 580–599 and 788 (for the period 1 December 1995 to 30 June 1999) or the Tenth Revision (ICD-10) codes: N00–N39 (for the period 1 July 1999 to 31 December 2016). The diagnosis of cause-specific urologic diseases includes the subcategories of: (i) kidney disease N00–N19, N25–N27 (ICD-10 codes) and 580–591 (ICD-9 codes); (ii) renal failure N17–N19 (ICD-10 codes) and 584–586 (ICD-9 codes); (iii) urolithiasis N20–N23 (ICD-10 codes) and 592 (ICD-9 codes); (iv) urinary-tract infection N39 (ICD-10 codes) and 599 (ICD-9 codes). We stratified the data by sex (male and female) and age (0–59, 60–74, ≥75 years old).

During the study period, Queensland was divided into 443 postal areas. Postal areas approximate postcodes and are created by the Australian Bureau of Statistics (ABS). We stratified the hospitalization data by socio-economic status according to the ABS’s Socio-Economic Indexes for Areas Index of Relative Socio-Economic Advantage/Disadvantage (IRSAD) in order to summarize the relative advantage, or disadvantage, of a neighbourhood.^14^ IRSAD summarizes information about the economic and social conditions of people and households within an area, including both relative advantage and disadvantage measures. A high IRSAD score generally means a relative lack of disadvantage and greater advantage. For example, an area with a high score means many households with high incomes, or many people in skilled occupations, or both. From these scores, the Queensland population was divided into three socio-economic status areas from high to low (144 high-score postal areas, 145 middle-score postal areas and 146 low-score postal areas) (Supplementary Figure S1, available as Supplementary data at *IJE* online).

Daily meteorological data on minimum, maximum temperatures and relative humidity were obtained from the Scientific Information for Land Owners (SILO) hosted by the Science and Technology Division of Queensland Government’s Department of Environment and Science (www.des.qld.gov.au/). The data sets are an observationally based data set constructed from station data interpolated to a 5 × 5 km grid. SILO uses mathematical interpolation techniques to construct spatial grids and infill gaps in time-series data sets.^15^ Daily mean temperatures were calculated as the mean of daily maximum and minimum temperatures. Daily meteorological data were linked to hospitalizations according to date and postal area (the average value of all grids covering the postal area). We trisected the postal areas according to the 21 years’ average mean temperatures from high to low, i.e. 148 hot-climate postcode areas (temperature ranges: −4.0°C∼48.4°C), 147 moderate-climate postcode areas (temperature ranges: −4.5°C∼48.0°C) and 148 cold-climate postcode areas (temperature ranges: −8.9°C∼46.8°C) (Supplementary Figure S2, available as Supplementary data at *IJE* online).

In this study, because our primary interest was the heat effect, all the data were restricted to the hot season, defined as the hottest four months of Queensland, which are December–March. For example, December 1995 to March 1996 was defined as the hot season in 1996.

### Statistical analysis

#### Temperature–hospitalization associations

A time-stratified, case-crossover design fitted with a conditional quasi-Poisson regression model was used to estimate the relative risks (RRs) of daily hospitalizations for urologic disease associated with temperature during the hot season.^16^ A time-stratified, case-crossover study was constantly used to analyse the acute effect of environmental exposure on health.^17^ By comparing case days with control days, some individual-level variants and time-invariant confounders are controlled for automatically. The primary statistical model is as follows:

(1) $$\;\mathrm{Log}\;(P_{it})=\alpha +\beta *{\mathrm{stratum}}_{it}+cb({\mathrm{temp}}_{it})+ns\left({rh}_{it}\right)+\mathrm{holiday}$$

*P_it_* represents the counts of hospitalization in postcode *t* on day *i*; *α* is the intercept. We created a categorical variable that combined year, month, day of the week and postcode together, to define the *stratum_it_*. Each calendar month in the same year and the same postcode was defined as a stratum. The cases and controls are matched by day of the week in the stratum. We assumed that there were no random or systematic between-city effects in this study. *cb*(*temp_it_*) is a 2D (exposure–response and lag–response dimensions) cross-basis function to model the lagged associations between daily mean temperature and hospitalizations. There is a linear relationship between temperature and hospitalizations for renal diseases in the hot season, so we used a linear function for the temperature–response dimension, with 0–10 lag days and a natural cubic spline with three degrees of freedom (*df*) for lag dimension in the cross-basis function.^18^^,^^19^*ns*(*rh_it_*) represents the natural cubic spline function of the daily mean relative humidity with three *df*. As suggested by previous studies,^20^^,^^21^ we used a moving average relative humidity of 0–10 lag days and we set the knots at the 25th and 75th percentiles of the distribution of humidity. The holiday variable is binary.

The heat effect implies the increased relative risk of hospitalizations for renal disease per 1°C increase in daily average temperatures. The intragroup difference was checked using meta-regression.^22–24^ For example, the heat-effect difference between females and males was examined by setting a postcode-specific coefficient in females and males as the dependent variable and the gender as a binary predictor, weighted by the inverse of the variance of the coefficient estimates.

#### Attributable hospitalizations

The number of hospitalizations attributable to heat exposure was estimated using the formula:^25^

(2) DANi = (RRi–1)/RRi × Ni

*DANi* is the number of hospitalized cases attributable to heat exposure on day *i*; *RRi* is the relative risk of hospitalizations for renal diseases per 1°C increase in daily mean temperatures on day *i*. *Ni* is the average number hospitalized in the 10 days following day *i*. The specific causes of renal diseases were calculated in the same way. The attributable fraction (*AFi*) equates to (*RRi*-1)/*RRi*. The total number of attributable hospitalizations (*ANi*) is calculated by *AFi* multiplying the total number of hospitalizations during the study period. As with previous studies, the 95% CIs for the attributable numbers were calculated from the 95% CIs of the RRs using Monte Carlo simulations.^25^^,^^26^

### Sensitivity analyses

The non-linear function was used for temperature variables in sensitivity analyses to assess whether the linear function could accurately predict the temperature–hospitalization relationship. A sensitivity test using meta-regression was performed by changing the temperature lag from 0–8 to 0–12 days and the *df* of lag days from three to five to check whether the original model was robust enough. We used a lag–response curve to verify whether a 10-day lag was enough to capture the heat effects.

All data analyses are conducted using R software (version 3.5.1). The ‘dlnm’ package can be used to fit the linear lagged effect of daily mean temperature, the ‘gnm’ package to fit the conditional quasi-Poisson regression. The ‘mvmeta’ package was used for the meta-regression analysis.

## Results

**Figure 1:**
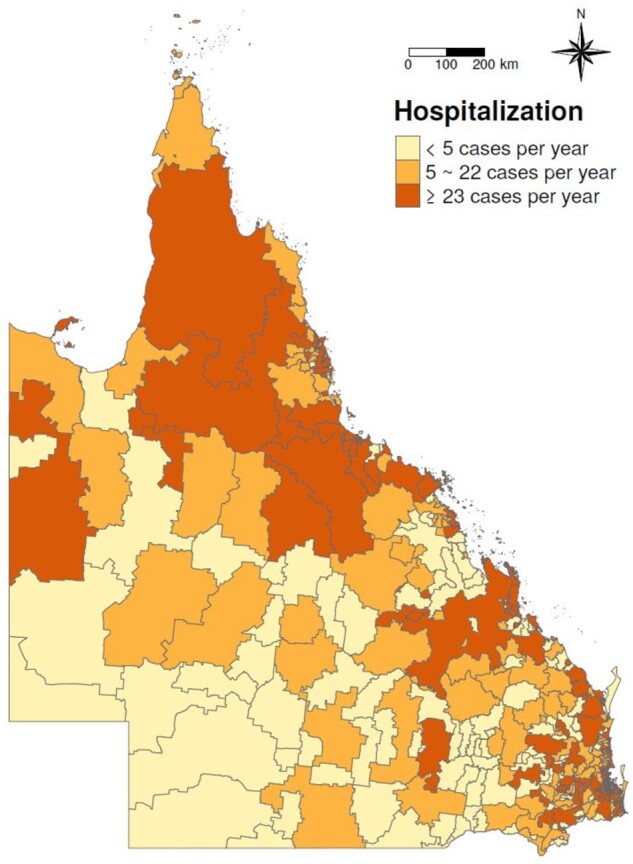
Annual distribution of average number of hospitalized cases for urologic diseases in Queensland, Australia, during the 1995–2016 hot seasons

The descriptive results are shown in Table 1. A total of 238 427 hospital admissions for urologic diseases were recorded between the four hot-season months in the period between 1995 and 2016. The sex ratio was 1.08 male:1.00 female. The average daily mean hot-season temperature and relative humidity were 25.2°C (17.9°C–33.5°C) and 71.1%, respectively. The annual average distribution of hospitalizations for urologic diseases is shown in Figure 1. The detailed information about the enrolled hospitalized cases of cause-specific urologic diseases is shown in Supplementary Table S1 (available as Supplementary data at *IJE* online).

**Table 1:** Distribution of enrolled hospitalizations and temperature features in the 443 postal areas between the 1995 and 2016 hot seasons in Queensland, Australia

	Subgroup	No. of cases	Average postal area temperatures (°C)			Average postal area relative humidity
			Mean	Minimum	Maximum	Mean
	Total	238 427	25.2	17.9	33.5	71.1%
Climate						
	Hot	47 693	27.1	19.9	33.7	66.6%
	Mild	104 041	24.7	17.9	33.8	72.8%
	Cold	86 693	23.9	15.9	33.1	73.9%
IRSAD						
	Low	83 370	25.3	17.9	32.9	71.7%
	Middle	84 616	25.6	17.9	33.8	69.5%
	High	70 419	24.8	17.8	34.0	53.0%
Sex						
	Men	123 788				
	Women	114 639				
Age (years)						
	0–59	120 733				
	60–74	61 773				
	75+	55 921				

Climate regions were divided according to the daily mean temperature of the postal areas. IRSAD, Index of Relative Socio-Economic Advantage/Disadvantage.

The associations between heat exposure and hospitalizations for urologic diseases over lag 0–10 days among different subgroups are shown in Table 2. At the state level, a 1°C increase in daily mean temperature was associated with a 3.3% (95% CI: 2.9%, 3.7%) increase in hospitalizations for urologic diseases. The heat effect was marginally stronger (*p*-value = 0.05) for males compared with females, with increased hospitalization risks of 3.9% (95% CI: 3.4%, 4.3%) and 2.6% (95% CI: 2.2%, 3.1%), respectively. However, this difference was not statistically significant. The estimated risks of hospitalization for urologic diseases increased along with age. We observed a significant difference in heat effects between individuals ≥60 years old and individuals <60 years old. The heat effects are 2.5% (95% CI: 2.1%, 3.0%) in the age group ≤59 years old, 3.9% (95% CI: 3.4%, 4.4%) in those aged 60–74 years and 4.3% (95% CI: 3.8%, 4.8%) in those aged ≥75 years old. We did not find significant differences in heat effects according to climate zones or socio-economic status.

**Table 2:** Cumulative associations between heat exposure (1°C increase in daily mean temperatures) and hospitalizations over lag 0–10 days stratified by age, sex, climate zone and socio-economic status groups

		Percentage increase of hospitalization (95% CI)	*P*-value
Total	Total	3.3% (2.9%, 3.7%)	
Sex	Female	2.6% (2.2%, 3.1%)	Reference
	Male	3.9% (3.4%, 4.3%)	0.05
Age(years)	0–59	2.5% (2.1%, 3.0%)	Reference
	60–74	3.9% (3.4%, 4.4%)	0.04
	75+	4.3% (3.8%, 4.8%)	0.01
Climate	Cold	3.0% (2.3%, 3.6%)	0.36
	Mild	3.8% (3.1%, 4.5%)	Reference
	Hot	2.9% (2.1%, 3.7%)	0.38
IRSAD	Low	3.0% (2.3%, 3.7%)	0.30
	Middle	4.0% (3.3%, 4.6%)	Reference
	High	2.9% (2.2%, 3.6%)	0.29

Differences between groups were tested by meta-regression. *P*-value < 0.05 means significant difference.

**Figure 2:**
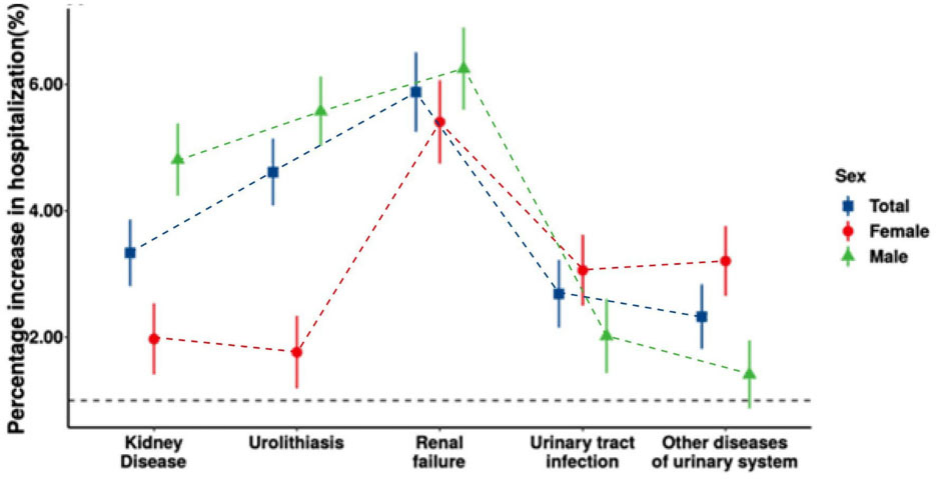
Cumulative associations between heat exposure (1°C increase in daily mean temperatures) and hospitalizations over lag 0–10 days stratified by different sex of cause-specific urologic diseases

Figure 2 shows the cumulative associations between heat exposure and hospitalizations for cause-specific urologic diseases and different sex groups over lag 0–10 days between 1995 and 2016. The percentage change of hospitalizations per 1°C increase in mean temperatures is shown in Supplementary Table S2 (available as Supplementary data at *IJE* online). The strongest association with heat exposure was found in renal failure. The hospitalizations for renal failure increased by 5.88% (95% CI: 5.25%, 6.51%). We noticed a sex-specific pattern in different diseases. Males demonstrated significantly stronger (*p*-value < 0.001) associations with heat exposure in kidney disease and urolithiasis than females. There was no sex difference in the associations between heat exposure and hospitalizations for renal failure.

**Figure 3:**
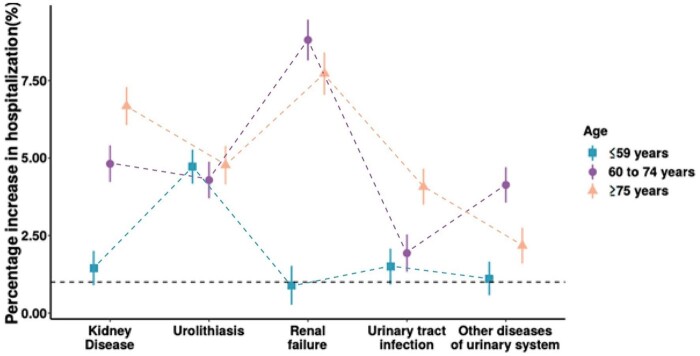
Cumulative associations between heat exposure (1°C increase in daily mean temperatures) and hospitalizations over lag 0–10 days stratified by different age groups of cause-specific urologic diseases

Figure 3 shows the associations between heat exposure and hospitalizations for cause-specific urologic diseases of different age groups over lag 0–10 days between 1995 and 2016. The percentage change in hospitalizations per 1°C increase in mean hot temperatures is shown in Supplementary Table S3 (available as Supplementary data at *IJE* online). The associations were stronger (*p*-value < 0.001) in the elderly (≥60-year-old) group than in the <60-year-old group in kidney disease and renal failure. The associations were stronger in the ≥75-year-old than the ≤74-year-old age group for urinary-tract infection. There was no difference in the associations between age and heat exposure and hospitalizations for urolithiasis.

Hospitalizations for urologic diseases attributable to hot temperatures are shown in Table 3. In total, 19.2% (95% CI: 17.2%, 21.2%) of hospitalizations can be attributable to high temperatures, which accounted for 45 700 (95% CI: 40 900 to 50 400) cases during the 21-year period of 4-month-long hot seasons. The highest attributable fraction was in the renal-failure group, with increased hospitalizations by 31.2% (95% CI: 28.5%, 33.7%). We observed distinct heat effects according to sex and age. The estimated attributable rate was higher in males [22.2% (95% CI: 20.0%, 24.3%)] than in females [15.8% (95% CI: 13.3%, 18.2%)] and higher in the ≥60-year-old than in the <60-year-old group.

**Table 3:** Hospitalizations attributable to heat exposure over lag 0–10 days in Queensland during the 1996–2016 hot seasons stratified by age, sex, climate zone, socio-economic status and cause-specific urologic diseases

	Group	Attributable cases (95% CI)	Attributable fraction (95% CI)
	Total	45 700 (40 900, 50 400)	19.2% (17.2%, 21.2%)
Climate			
	Hot	7540 (5650, 9330)	15.8% (11.9%, 19.6%)
	Mild	22 600 (19 000, 26 000)	21.7% (18.3%, 25.0%)
	Cold	15 900 (12 900, 18 700)	18.4% (14.9%, 21.7%)
IRSAD			
	Low	15 000 (12 000, 17 900)	18.0% (14.4%, 21.5%)
	Middle	19 000 (16 200, 21 600)	22.4% (19.2%, 25.5%)
	High	12 000 (9360, 14 500)	17.1% (13.3%, 20.7%)
Sex			
	Men	27 400 (24 700, 30 100)	22.2% (20.0%, 24.3%)
	Women	18 100 (15 200, 20 800)	15.8% (13.3%, 18.2%)
Age (years)			
	0–59	18 400 (15 400, 21 200)	15.2% (12.8%, 17.6%)
	60–74	13 700 (12 200, 15 200)	22.3% (19.8%, 24.7%)
	75+	13 500 (12 100, 14 800)	24.2% (21.7%, 26.6%)
Cause-specific			
	Kidney disease	12 300 (10 600, 14 000)	19.6% (16.8%, 22.2%)
	Renal failure	7060 (6450, 7640)	31.2% (28.5%, 33.7%)
	Urolithiasis	15 500 (14 000, 16 900)	25.8% (23.3%, 28.2%)
	Urinary-tract infection	9880 (8090, 11 600)	16.2% (13.3%, 19.1%)
	Other renal diseases	7470 (5950, 8940)	13.8% (11.0%, 16.5%)

The hot season in 1996 was the period December 1995 to March 1996.

The association between heat exposure and hospitalizations for urologic diseases was linear (Supplementary Figure S3, available as Supplementary data at *IJE* online). Our results were robust when changing the lag days of temperature from 0–8 to 0–12 (Supplementary Table S4, available as Supplementary data at *IJE* online) and the *df* of lag days from three to five (Supplementary Table S5, available as Supplementary data at *IJE* online). The lag–response curve showed that 10 days was enough to capture the heat effects (Supplementary Figure S4, available as Supplementary data at *IJE* online).

## Discussion

This research is the first subtropical–tropical area study to examine the demographic, geographic, socio-economic and cause-specific characteristics in the association between heat exposure and risk of hospitalizations for urologic diseases. We found that a 1°C increase in temperature in the hot season was associated with a 3.3% (95% CI: 2.9%, 3.7%) increase in hospitalization for urologic diseases. Males and the elderly (both sexes) (≥60 years old) showed stronger heat effects than females and the <60-year-old age group. The sex-specific and age-specific patterns with heat exposure were similar among cause-specific urologic diseases. In cause-specific urologic disease analysis, hospitalizations for renal failure showed the strongest associations with daily mean temperatures in the hot season. The attributable risk of renal failure was the greatest among the specific causes of urologic diseases. Our result demonstrated distinct sex- and age-specific patterns. Males and the elderly were more vulnerable to heat exposure than the corresponding populations. The evidence provided by this analysis supports a targeted public health-promotion campaign to raise heat awareness among these identified susceptible populations. It is vital to promote prevention strategies to reduce renal failure and related complications as the temperature warms due to climate change. This research finding supports a heat-warning system to prevent hospital admissions for urologic diseases early, especially for the elderly. It is of interest to note that within this ‘hot’ season, temperatures still included relatively ‘mild’ temperatures that ranged from 17.9°C.

The relationship between heat exposure and hospitalizations for urologic diseases observed in Queensland is similar to findings from population studies in other geographic locations. A study in California reported that for every 10°F (5.56°C) increase in the daily mean temperature in the warm season (May–October), the risk of hospital admissions for kidney diseases increased by 12.9% (95% CI: 8.7%, 17.3%) over lag 0–14 days during the period 1999–2009.^19^ The size of the estimated effects in our results (3.3% per 1°C for kidney disease) was stronger than those in that recent Californian study. Compared with those findings, the Queensland population appears to be more vulnerable to high temperatures, at least in terms of renal diseases. Another study conducted in New York reported an association between a 5°F (2.78°C) increase in the daily mean temperature and a 9% increase in the risk of hospitalizations for acute renal failure over lag 1 day during the hot season (July and August) in the period 1991–2004.^13^ In Australia, a study in Adelaide, a temperate city, found that a 1°C increase in daily average temperatures was associated with a 1% (95% CI: 0.7%, 1.3%) increase in urologic disease for patients during the warm season (October–March) during the period 2003–2014.^8^ The estimated effects of heat exposure in this study were stronger than in the studies conducted in New York, California and Adelaide.^8^^,^^13^^,^^19^^,^^27^ This finding is possibly due to the fact that the exposure assessment was based on postcodes and, at this finer resolution, we found stronger effects.

Numerous studies demonstrate that recurrent heat exposure and dehydration may cause renal hypoperfusion,^28^ tubule injury and inflammation.^29^ These diseases could lead to a reduced glomerular filtration rate and acute kidney injury.^30^ A recent study of 105 healthy and well-hydrated Guatemalan sugarcane workers found that declines in renal function were associated with high temperatures.^31^ Kidney injury caused by heat stress could be worsened by increasing core body temperatures.^32^ These epidemiological (and other animal) findings partly explain the positive associations between heat exposure and increased risks of hospitalizations for urologic diseases in our study.

The positive associations found in our study were modified by demographic factors. The elderly (≥60 years old) showed a larger increase in risk of hospitalization. Consistently with our findings, the study in Queensland found that people >64 years old were more vulnerable to heat in terms of emergency-department visits for acute kidney injury than those ≤64 years old.^9^ Prior studies indicated that the elderly have a reduced thermal perception, making them less sensitive to high temperatures.^33^ In addition, they have impaired thermoregulatory capacity^34^ and have attenuated the physiological ability to dissipate heat.^35^ Together with medication and physiologically degraded renal function, the elderly are more susceptible than younger age groups. However, the study in New York found that the increased susceptibility was strongest among the 25- to 44-year-old age group.^13^ This finding was attributed to their higher participation in outdoor activities during the hot season.^13^ Borg *et al.* found that those <65 years old had statistically significant results for emergency-department admissions for urolithiasis, renal failure and acute kidney injury (AKI), but this was not found for those ≥65 years old.^8^

In our analysis, males demonstrated a larger increase in the risk of hospitalizations for urologic disease than females, but the difference was not statistically significant. Consistently with this finding, Lim *et al.* found that the increased risk of admissions for AKI for males was greater than that for females per 1°C increase in the daily mean temperature in the warm season during the period 2007–2014 in Seoul, Korea.^27^ The higher association for males could possibly be due to behaviour patterns. Males tend to carry out more strenuous outdoor work during the hot season compared with females, resulting in higher duration and frequency of heat exposure.^36^ In villages in Central America, the indicators of decreased renal function were more common among males than females.^36^ In addition, sex hormones act differently in the progression of kidney disease. Males were found to have a higher progression rate of chronic kidney disease compared with females.^37^ Testosterone was associated with a reduction in renal function,^38^ whereas oestrogen appeared to play a protective role.^39^ The deleterious effects of hot temperatures on the renal system may be caused in part by male hormones.

The positive associations between heat exposure and hospitalizations for urologic disease were robust among all specific types of urologic diseases examined. The effect estimate for renal failure was larger than that for other urologic diseases tested in our study. Consistently with our results, the study in New York found that the risks of hospitalization for acute renal failure of a 5°F (2.78°C) increase in the daily mean temperature during their hot season of July and August for the period 1991–2004 at lag 1 day was larger than those for the other renal-system diseases.^13^ Another Californian study found that the risks of hospitalization for acute renal failure increased by 7.4% (95% CI: 4.0%, 10.9%) per 10°F (5.56°C) increase in the daily mean apparent temperature from May to September during the period 1999–2005 at lag 0. The heat effect of acute renal failure was larger than that for respiratory-system diseases and diabetes, but smaller than that for dehydration [10.8% (95% CI: 4.0%, 10.9%)].^40^ In a study from Illinois, one of the most common reasons for hospitalization was acute renal disorders during summertime per 1°C increase in the monthly maximum temperatures (May–September) for the period 1987–2014.^41^ Biomarkers of AKI even increased in individuals who walked three 20-minute sessions (4.8 km/h) in a 38°C, 50% relative humidity environment with a 10-minute standing rest in between walking sessions.^42^ All these findings confirm the sensitivity of kidney function to hot temperatures. All these assessments indicate that the increase in temperatures in the hot season exacerbated by climate change could worsen the change in renal-function indicators, leading to an increase in hospital admissions for renal failure.

We found a strong age-specific pattern in the heat–renal failure associations. The elderly (≥60-year-old age group) demonstrated stronger associations with heat in terms of renal failure than the young (<60-year-old age group). Our results warrant immediate investigation of a wide-ranging renal-failure screening campaign among the elderly at this location and consideration of other areas with similar characteristics in Australia.

The positive associations between heat exposure and hospitalizations for urolithiasis were consistent with previous findings in several different locations worldwide.^19^^,^^43^^,^^44^ Consistently with our results, Tasian *et al.* found positive associations between high daily temperatures (30°C) and kidney-stone presentations in five US cities over lag 20 days during the period 2005–2011.^43^ Our results demonstrated a clear sex-specific pattern. Risks of hospitalizations for urolithiasis were significantly stronger in males than in females. Consistently with this finding, Fakheri *et al.* demonstrated that the annual mean temperature was positively associated with the increasing prevalence of kidney stones, which was mainly found to affect males.^45^ A study in South Carolina found that the association between high temperature (99th percentile of daily wet-bulb temperatures) and emergency-room visits for nephrolithiasis was stronger in males than in females. This study also found that the stronger heat effect in males was probably due to physiologic differences rather than the exposure model.^46^ It is important to note that there was no age-specific difference in heat–urolithiasis associations in this study. This result suggests a common-sense finding that, to encourage urolithiasis prevention for all age groups, individuals should remove themselves from hot temperatures and stay hydrated.

In this study, we did not observe a significant difference in heat effects by climate zones or socio-economic status. Consistently with our results, a nationwide study in Brazil also found insignificant differences in heat–hospitalization associations for urologic diseases between lower-middle-income cities at 9.6% (6.2–13.1%) increase per 5°C increase in the daily mean temperature during the hot season and high-income cities at 4.9% (1.8–8.0%) increase per 5°C increase in the daily mean temperature during the hot season.^47^ This is inconsistent with the general impression that low socio-economic status reflects a low educational level, limited use of air conditioning or no health insurance, which may exacerbate the heat effect. The inconsistency is speculated to be modified by the difference in population characteristics and other country- or region-specific factors. Due to the lack of necessary information, we were unable to explore this research question and further studies are needed to clarify it.

The 21 years of historical data and large hospital-admissions sample size at the fine-grained spatial resolution of postal area allowed us to detect small size effects. However, this study has several limitations. We did not use other thermal indicators, such as minimum and maximum temperatures, to check their associations with hospitalizations from urologic diseases. Further, we were unable to consider indoor temperature exposure due to the lack of data. Neither did we analyse other cause-specific urologic diseases or consider other environmental and clinical factors due to a lack of data.

## Conclusions

Exposure to high temperatures was associated with an increase in hospitalizations for urologic disease in Queensland during the hot season (December–March) in the period 1995–2016. The positive associations were robust among all subgroups. Stronger heat effects were found for males and the elderly compared with females, and young males or females. Hospitalizations for renal failure demonstrated the strongest associations with heat exposure. Our results support the suggestion that public health-promotion campaigns should be launched now to raise heat awareness among susceptible populations. Specifically, annual kidney-disease screening programmes are recommended for the elderly to detect early urologic diseases.

## Supplementary data

Supplementary data are available at *IJE* online.

## Ethics approval

Ethics approval is not needed because all the subjects in this study are anonymous.

## Funding

This study was supported by the Australian National Health & Medical Research Council [#APP2000581] and the Taishan Scholar Program. Y.G. was supported by a Career Development Fellowship of the Australian National Health and Medical Research Council (#APP1107107 and #APP1163693). S.L. was supported by an Early Career Fellowship of Australian National Health and Medical Research Council [#APP1109193].

## Data availability

The data underlying this article will be shared on reasonable request to the corresponding author.

## Author contributions

P.L.: formal analysis, investigation, methodology, software, validation, visualization, writing—original draft, writing—review and editing; G.X.: formal analysis, investigation, methodology, software, validation, visualization, writing—original draft, writing—review and editing; Q.Z.: data curation, investigation, review and editing; D.G.: investigation, review and editing; Y.-H.L.: investigation, review and editing; S.L.: conceptualization, investigation, methodology, validation, review and editing, funding acquisition, project administration, resources, supervision; Y.G.: conceptualization, data curation, investigation, methodology, validation, review and editing, funding acquisition, project administration, resources, supervision.

## Conflict of interest

None declared.

## Supplementary Material

dyab189_Supplementary_Data
