# Supplementary material for: Attributable risks of hospitalizations for urologic diseases due to heat exposure in Queensland, Australia, 1995–2016
Source: Int J Epidemiol. 2021 Sep 11;51(1):144–54. doi: 10.1093/ije/dyab189 (PMC8855997; doi:10.1093/ije/dyab189)
Supplement: dyab189_Supplementary_Data [file dyab189_supplementary_data.docx]

**Supplementary Materials**


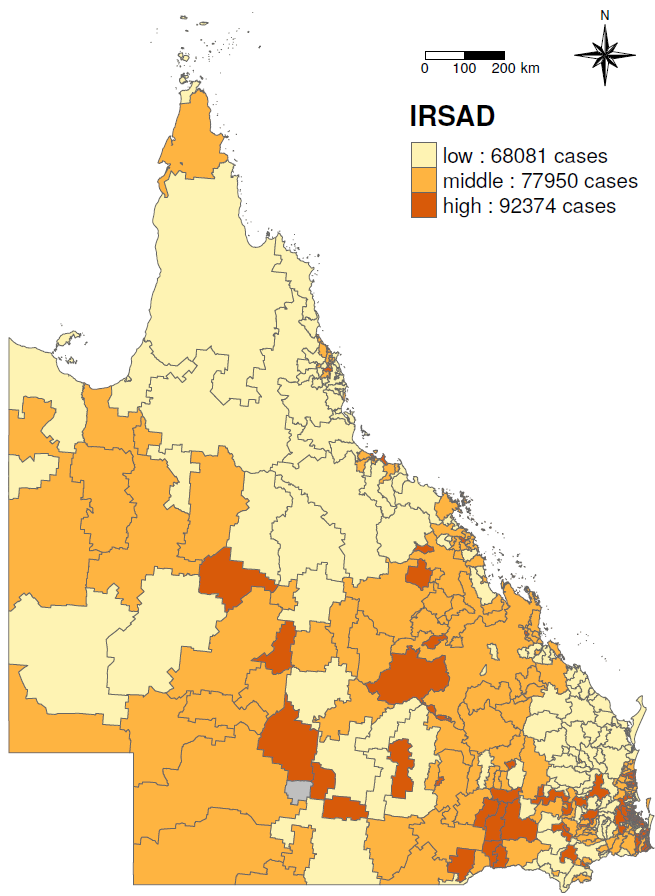


Figure S1 Distribution of different Index of Relative Socio-Economic Advantage/Disadvantage in Queensland, Australia

Note: Grey area indicates that there is no validated IRSAD data.


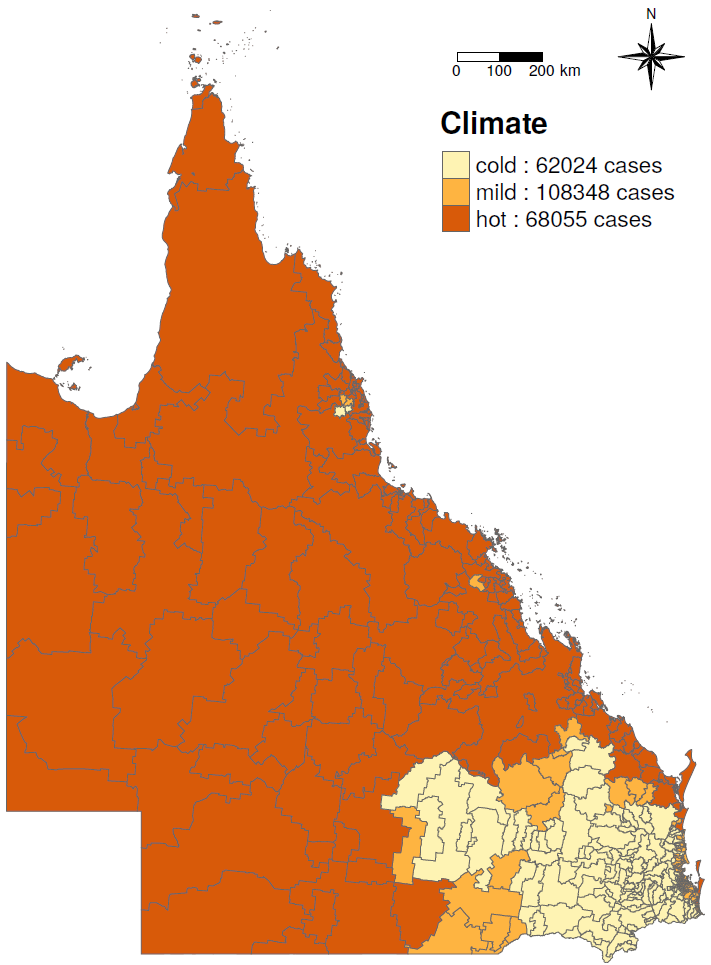


Figure S2 Distribution of different climate zones (trisected the postal areas according to the 21 years’ average mean temperatures from high to low) in Queensland, Australia.

Note: Hot climate zones (148 hot climate postcode areas), Mild climate zones (147 moderate climate postcode areas), cold climate zones (148 cold climate postcode areas)

Table S1 Hospitalized cases of cause-specific urologic diseases among different subgroups between 1995 and 2016 hot season, in Queensland, Australia.

|  |  | Kidney disease | Renal failure | Urolithiasis | Urinary tract infection | Other |
| --- | --- | --- | --- | --- | --- | --- |
|  | Total | 63147 | 22695 | 60119 | 61038 | 54123 |
| Sex | Female | 32572 | 9955 | 15421 | 39114 | 27532 |
|  | Male | 30575 | 12740 | 44698 | 21924 | 26591 |
| Age | 0-59 | 34996 | 7094 | 39347 | 22148 | 24242 |
|  | 60-74 | 14770 | 6579 | 16281 | 12235 | 18487 |
|  | ≥75 | 13381 | 9022 | 4491 | 26655 | 11394 |

Table S2 Percentage increase of hospitalizations for cause-specific urologic diseases per 1℃ increase in daily mean temperatures over 0-10 days stratified by sex.

| Cause-specific diseases | Subgroups | Percentage increase of hospitalization | p value |
| --- | --- | --- | --- |
| Kidney | Total | 3.34% (2.81%, 3.87%) |  |
|  | female | 1.97% (1.41%, 2.54%) | ref |
|  | male | 4.81% (4.24%, 5.38%) | <0.01 |
| Renal Failure | Total | 5.88% (5.25%, 6.51%) |  |
|  | female | 5.41% (4.75%, 6.07%) | ref |
|  | male | 6.25% (5.60%, 6.90%) | 0.36 |
| Urolithiasis | Total | 4.61% (4.09%, 5.14%) |  |
|  | female | 1.76% (1.19%, 2.34%) | ref |
|  | male | 5.57% (5.03%, 6.12%) | <0.01 |
| Urinary tract infection | Total | 2.69% (2.15%, 3.22%) |  |
|  | female | 3.06% (2.50%, 3.62%) | ref |
|  | male | 2.02% (1.43%, 2.61%) | 0.20 |
| Other | Total | 2.33% (1.81%, 2.84%) |  |
|  | female | 3.21% (2.66%, 3.76%) | ref |
|  | male | 1.41% (0.87%, 1.95%) | 0.02 |

Note: differences between groups were tested by meta-regression. P<0.05 means there were significant difference

Table S3 Percentage increase of hospitalizations for cause-specific urologic diseases per 1℃ increase in daily mean temperatures over 0-10 days stratified by age.

| Cause-specific | subgroups | Percentage increase of hospitalizaton | P value |
| --- | --- | --- | --- |
| kidney | age0to59 | 1.45% (0.89%, 2.01%) | ref |
|  | age60to74 | 4.82% (4.23%, 5.41%) | <0.01 |
|  | age75 | 6.68% (6.06%, 7.30%) | <0.01 |
| urolithiasis | age0to59 | 4.72% (4.17%, 5.27%) | ref |
|  | age60to74 | 4.29% (3.70%, 4.88%) | 0.59 |
|  | age75 | 4.77% (4.15%, 5.39%) | 0.95 |
| Renal failure | age0to59 | 0.90% (0.27%, 1.53%) | ref |
|  | age60to74 | 8.81% (8.15%, 9.47%) | <0.01 |
|  | age75 | 7.72% (7.04%, 8.41%) | <0.01 |
| uti | age0to59 | 1.50% (0.92%, 2.08%) | ref |
|  | age60to74 | 1.93% (1.33%, 2.54%) | 0.60 |
|  | age75 | 4.07% (3.49%, 4.66%) | <0.01 |
| other | age0to59 | 1.12% (0.58%, 1.66%) | ref |
|  | age60to74 | 4.13% (3.56%, 4.71%) | <0.01 |
|  | age75 | 2.17% (1.60%, 2.75%) | 0.18 |

Note: differences between groups were tested by meta-regression. P<0.05 means there were significant difference


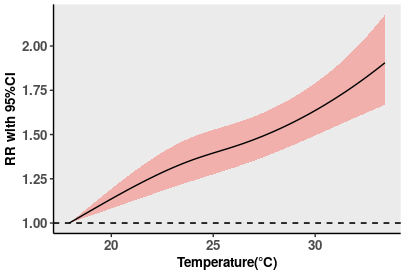


Figure S3 The association between heat exposure and hospitalizations for urologic diseases with non-linear function of temperatures

Table S4 The associations between heat exposure and hospitalizations of urologic disease when changing lag days of temperature from 0-8 to 0-12

| model | p-value |
| --- | --- |
| Lag 0-8 days | 0.13 |
| Lag 0-9 days | 0.48 |
| Lag 0-10 days | reference |
| Lag 0-11 days | 0.92 |
| Lag 0-12 days | 0.46 |

Table S5 The associations between heat exposure and hospitalizations of urologic disease when changing df of lag days from three to five

| model | p-value |
| --- | --- |
| df3 | reference |
| df4 | 0.98 |
| df5 | 0.99 |


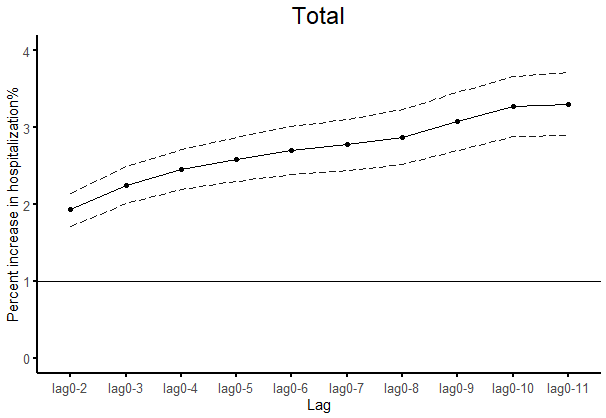


Figure S4 The lag-response curve of the heat effects
